# Two Genetically Similar H9N2 Influenza A Viruses Show Different Pathogenicity in Mice

**DOI:** 10.3389/fmicb.2016.01737

**Published:** 2016-11-04

**Authors:** Qingtao Liu, Yuzhuo Liu, Jing Yang, Xinmei Huang, Kaikai Han, Dongmin Zhao, Keran Bi, Yin Li

**Affiliations:** ^1^Key Laboratory of Veterinary Biological Engineering and Technology, National Center for Engineering Research of Veterinary Bio-products, Institute of Veterinary Medicine, Ministry of Agriculture, Jiangsu Academy of Agricultural SciencesNanjing, China; ^2^Jiangsu Key Laboratory of Zoonosis, Jiangsu Co-Innovation Center for Prevention and Control of Important Animal Infectious Diseases and ZoonosesYangzhou, China

**Keywords:** H9N2, influenza A virus, genetic background, pathogenicity, mice

## Abstract

H9N2 Avian influenza virus has repeatedly infected humans and other mammals, which highlights the need to determine the pathogenicity and the corresponding mechanism of this virus for mammals. In this study, we found two H9N2 viruses with similar genetic background but with different pathogenicity in mice. The A/duck/Nanjing/06/2003 (NJ06) virus was highly pathogenic for mice, with a 50% mouse lethal dose (MLD_50_) of 10^2.83^ 50% egg infectious dose (EID_50_), whereas the A/duck/Nanjing/01/1999 (NJ01) virus was low pathogenic for mice, with a MLD_50_ of >10^6.81^ EID_50_. Further studies showed that the NJ06 virus grew faster and reached significantly higher titers than NJ01 *in vivo* and *in vitro*. Moreover, the NJ06 virus induced more severe lung lesions, and higher levels of inflammatory cellular infiltration and cytokine response in lungs than NJ01 did. However, only 12 different amino acid residues (HA-K157E, NA-A9T, NA-R435K, PB2-T149P, PB2-K627E, PB1-R187K, PA-L548M, PA-M550L, NP-G127E, NP-P277H, NP-D340N, NS1-D171N) were found between the two viruses, and all these residues except for NA-R435K were located in the known functional regions involved in interaction of viral proteins or between the virus and host factors. Summary, our results suggest that multiple amino acid differences may be responsible for the higher pathogenicity of the NJ06 virus for mice, resulting in lethal infection, enhanced viral replication, severe lung lesions, and excessive inflammatory cellular infiltration and cytokine response in lungs. These observations will be helpful for better understanding the pathogenic potential and the corresponding molecular basis of H9N2 viruses that might pose threats to human health in the future.

## Introduction

Avian influenza A viruses (AIVs) of the H9N2 subtype were first detected in turkeys in the United States in 1966 (Homme and Easterday, 1970), and have been circulating worldwide in multiple avian species and endemic in poultry populations across Eurasia (Alexander, 2000, 2007; Perk et al., 2006; Bi et al., 2010; Fusaro et al., 2011). Of note, H9N2 viruses in poultry have occasionally been transmitted to humans and other mammals (Peiris et al., 2001; Butt et al., 2005, 2010; Sun et al., 2013), such as pigs and dogs, since this subtype was first reported to be detected in patients with influenza-like illness in Guangdong Province and in pigs in Hong Kong of China in 1998 (Guo et al., 1999; Lin et al., 2000; Peiris et al., 2001). In fact, follow-up serological surveys suggest that the incidence of human infections with H9N2 viruses might be more prevalent than what has been reported and possible human-to-human transmission cannot be completely excluded (Butt et al., 2005; Jia et al., 2008; Wang et al., 2009; Panwen et al., 2012). Clinically, human H9N2 infections present as typical seasonal influenza infections that can easily be overlooked (Lin et al., 2000; Butt et al., 2005), providing the viruses a greater opportunity to adapt to humans. These observations raise concerns about the possibility that H9N2 viruses might increase pathogenicity and transmissibility in humans. It is therefore important to investigate the pathogenicity and the corresponding mechanism of H9N2 viruses for mammals.

Previous studies revealed that some H9N2 viruses isolated from land-based poultry have demonstrated increased virulence for mammals. Guo et al. (2000) reported that the A/Chicken/Hong Kong/G9/97 and A/Quail/Hong Kong/G1/97 viruses could cause the deaths of three and two of the eight tested mice, respectively, at a dose of 10^6^ 50% egg infectious dose (EID_50_) in mice, while Lu et al. reported that the two viruses are not lethal for mice at the same dose (Choi et al., 2004). In another study, Li et al analyzed 27 representative H9N2 viruses isolated from chickens and ducks in Mainland China, and found that some chicken isolates were able to replicate in mouse lungs efficiently and could induce a 10–20% weight loss of the inoculated mice, but none of the viruses are lethal for mice (Li et al., 2005). However, in 2007–2009, Bi et al. (2010) isolated six H9N2 viruses from chickens in northern China and found that these viruses could cause 50–85.7% mortality in mice at a dose of 10^6^ EID_50_. In addition, an H9N2 virus isolated from guinea fowl also showed enhanced replication and efficient transmission by direct contact in a ferret model (Wan et al., 2008). Although all these viruses cause death in mice at a high dose, none of the viruses are highly pathogenic for mice according to the criteria that a highly pathogenic virus has a 50% mouse lethal dose (MLD_50_) value less than 10^3.0^ EID_50_ (Katz et al., 2000). However, several experimental evolutions by serial passage in mouse lungs showed that non-lethal H9N2 isolates could evolve to be lethal or highly pathogenic for mice after serial passage in mouse lungs (Zhang et al., 2011; Wang et al., 2012; Liu et al., 2014). Therefore, it is necessary to investigate the pathogenic potential and the corresponding molecular basis of H9N2 avian influenza viruses in mammals.

Several molecular determinants have already been identified that govern the pathogenicity of avian influenza virus for mammals, such as amino acid substitutions in the ribonucleoprotein (RNP) complex (Gabriel et al., 2005; Salomon et al., 2006; Song et al., 2009; Sun et al., 2015), the mutations involved in the ability of NS1 proteins to restrict the induction of the host interferon response (Li et al., 2006; Thulasi Raman and Zhou, 2016), the length of the NA stalk (Zhou et al., 2009). However, most of these studies focused on H5 subtype of influenza viruses, and the pathogenic mechanism of H9N2 viruses for mammals is poorly understood. The substitution PB2 E627K which has been shown to be a key factor in the increased virulence of H5N1 AIVs to mammals has also been observed in the adaption of H9N2 viruses in mice (Zhang et al., 2011; Wang et al., 2012). However, Wang et al. (2012) reported that although the E627K mutation on its own enhanced replication and polymerase activity, it did not significantly increase pathogenicity of H9N2 virus, and only the combination of PB2 E627K and M147L could increase the virulence of the H9N2 virus in mice. Another report also showed that a H9N2 virus containing a human-like PB2 segment with 627 K is non-pathogenic for mice, while the mutation F404L in the PB2 segment could increase the virulence of the H9N2 virus and the combination of PB2 F404L with mutations in PA (D3V and S225R) and HA (L80F and N193D) was able to make the non-pathogenic H9N2 virus become high pathogenic for mice (Liu et al., 2015). However, it is still unknown that if H9N2 virus could acquire the mutations that govern the high pathogenicity for mice in natural, especially in poultry.

In this study, we characterized two H9N2 AIVs, NJ06 and NJ01, that were isolated from ducks in China. The NJ06 virus was highly pathogenic for mice and induced severe lung lesions and excessive cytokine responses, while the NJ01virus exhibited low pathogenicity in this model. However, there were only twelve amino acid differences between the two viruses, which might contribute to the high virulence of the NJ06 virus in mice. Therefore, the two viruses had similar genetic background, but showed different pathogenicity for mice, which offer an appropriate system in which to explore the molecular basis of host adaptation and enhanced virulence in mammals.

## Materials and Methods

### Ethics Statements

All animal experiments were approved by the Committee on the Ethics of Animal Experiments of Jiangsu Academy of Agricultural Sciences (JAAS no. 20141107), and complied with the guidelines of Jiangsu Province Animal Regulations (Government Decree No. 45). All experiments involving live viruses and animals were carried out in negative pressure isolators with HEPA filters in a biosafety level 2+ laboratory (enhanced animal biosafety level 2 laboratory and a negative pressure-ventilation laboratory) in accordance with the institutional biosafety manual.

### Viruses and Cells

The H9N2 viruses A/duck/Nanjing/06/2003 (NJ06) and A/duck/Nanjing/01/1999 (NJ01) were isolated from ducks in Jiangsu, China and propagated in specific pathogen-free (SPF) embryonated chicken eggs. Viral titers were measured by calculating the EID_50_. Madin–Darby canine kidney (MDCK) cells were cultured in Dulbecco’s modified Eagle’s medium (DMEM) supplemented with 5% fetal bovine serum.

### Mouse Studies

Female BALB/c mice (5 weeks old) were used in this study. To evaluate the virulence of the NJ06 and NJ01 viruses, groups of five BALB/c mice were anesthetized with pentobarbital natricum and inoculated intranasally with 10-fold serial dilutions of viruses in 30 μl PBS or mock inoculated with PBS to serve as controls. Body weight and survival of mice were recorded daily for 14 days. Mice that showed severe symptoms or lost more than 25% of their body weight were euthanized and scored as dead for humane reasons. The MLD_50_ of virus was calculated and expressed in EID_50_.

To evaluate viral replication in mice, groups of female BALB/c mice were intranasally inoculated with 10^5^ EID_50_ of the NJ01 and NJ06 viruses, respectively. At 1, 2, 3, and 5 days post inoculation (dpi), five mice in each group were euthanized, and whole lungs were removed and homogenized in 1 ml of PBS for virus titration in 10-day-old embryonated eggs as previously described (Hu et al., 2013).

To assess lung injury, groups of five BALB/c mice were intranasally inoculated with 10^5^ EID_50_ of the NJ01 and NJ06 viruses, respectively, and lung histopathology and water content were determined at 5 dpi. For histopathological analysis, mouse lungs were fixed in 4% paraformaldehyde, embedded in paraffin, cut into 5 mm-thick sections and then stained with haematoxylin and eosin (H&E) for light microscopy. For water content analysis, mouse lungs were surgically dissected, blotted dry, and weighed immediately as wet weight, and then dried in an oven at 80°C for 72 h and reweighed as dry weight. The lung wet/dry weight ratios were calculated for each animal to assess tissue edema as previously described (Lang et al., 2005).

### Growth Properties *In vitro*

To evaluate the replication of virus *in vitro*, MDCK or A549 cell monolayer in 12-well plates were washed three times with PBS, and inoculated at a multiplicity of infection of 0.01, overlaid with serum-free DMEM containing 2 mg/ml TPCK-trypsin (Sigma–Aldrich). Virus titers in supernatants were determined as the number of 50% tissue culture infectious doses (TCID_50_) per ml in MDCK cells at 12, 24, 48, and 72 h post inoculation (hpi).

### Differential Leukocyte Counts and Cytokine Expression Analysis

Inflammatory response in mouse lungs was assessed by testing differential leukocyte counts in bronchoalveolar lavage (BAL) fluid and expression profiles of representative cytokine genes of mice infected with 10^5^ EID_50_ of the NJ06 or NJ01 virus at the indicated days. To determine differential leukocyte counts, BAL cells were obtained from mouse lungs in each group as described by Nick et al. (2000) and Densmore et al. (2013). In brief, the lungs were lavaged twice with a total 1 ml saline (4°C) through the endotracheal tube, and the recovery rate of BAL fluid was not less than 90% for each animal tested. After the amount of fluid recovered was recorded, an aliquot of BAL fluid was diluted 1:1 with 0.01% crystal violet dye and 2.7% acetic acid for leukocyte staining and erythrocyte hemolysis, and the number of leukocytes in BAL fluid was counted with a haemocytometer under a light microscope. Subsequently, the remaining fluid was centrifuged for 10 min at 300 × *g*. Cell differential counts were determined by Wright staining of a spun sample, on the basis of morphological criteria under a light microscope with evaluation of at least 200 cells per slide, and each slide was counted twice by different observers blinded to the status of the animal.

Quantitative real-time PCR (qRT-PCR) was used to analyze the expression of cytokine genes in mouse lungs. Total RNA was isolated from lungs using TRIzol reagent (Life Technologies) and treated with DNase I (Fermentas, Glen Burnie, MD, USA). One microgram of total RNA per sample was reverse transcribed into cDNA using a PrimeScript RT Reagent Kit (Takara). The cDNA was run in the ABI 7500 Real Time PCR System using an SYBR Premix Ex Taq Kit (Takara). One cycle for melting curve analysis for all reactions was added to verify product specificity. The expression of each cytokine gene relative to that of the β-actin was calculated using the 2*^-ΔΔCT^* method. The primers for TNF-α, CXCL10, IL-17a, and IL-10 were designed based on these target mouse genes with GenBank accession numbers NM_013693.3, NM_021274.2, NM_010552.3, and NM_010548.2, respectively. The primers for β-actin have been described previously (Hu et al., 2013). Primers for theses target genes were as follows (forward and reverse primers, respectively): for TNF-α, 5′-GCCAGGAGGGAGAACAGAAACTC-3′ and 5′-GGCCAGTGAGTGAAAGGGACA-3′; for CXCL10, 5′-ATCCGGAATCTAAGACCATCAAGAA-3′ and 5′-TGTCCATCCATCGCAGCAC-3′; for IL-17a, 5′-GAAGG CCCTCAGACTACCTCAA-3′ and 5′-TCATGTGGTGGTCCAGCTTTC-3′; for IL-10, 5′-GCCAGAGCCACATGCTCCTA-3′ and 5′-GATAAGGCTTGGCAACCCAAGTAA-3′; and for β-actin, 5′-CATCCGTAAAGACCTCTATGCCAAC-3′ and 5′-ATGGAGCCACCGATCCACA-3′.

### Sequence Analysis

Viral RNA was extracted from infected allantoic fluid using the Body Fluid Viral DNA/RNA Kit (Axygen) according to the manufacture’s protocol. Reverse transcription was performed using the Uni12-primer (5′-AGCAAAAGCAGG-3′) by standard methods, and PCR amplification of cDNA was directed with previously described primers (Hoffmann et al., 2001). PCR products were purified using a DNA Gel Extraction Kit (Axygen) in accordance with manufacture’s recommendations, and cloned into the pMD-18T vector (Takara), and sent for commercial sequence analysis (Sangon Biotechnology, Shanghai, China). Sequencing results were phylogenetically analyzed with the representative strains available in GenBank. The nucleotide sequences were initially aligned using the Clustal V alignment algorithm of the Megalign program (DNAStar, Madison, WI, USA). The phylogenetic tree was constructed using MEGA 6.06 software with the neighbor-joining method. The GenBank accession numbers for theNJ01 segments are KX349960, KX349961, DQ681205, and DQ681221 to DQ681225, and those for the NJ06 segments are KX349952 to KX349959.

### Statistical Analysis

Data were analyzed using the SPSS Statistics software and results were expressed as means ± standard deviation (SD). The statistical significance of differences was determined by an independent-sample *t*-test.

## Results

### Pathogenicity of NJ01 and NJ06 Viruses in Mice

**FIGURE 1:**
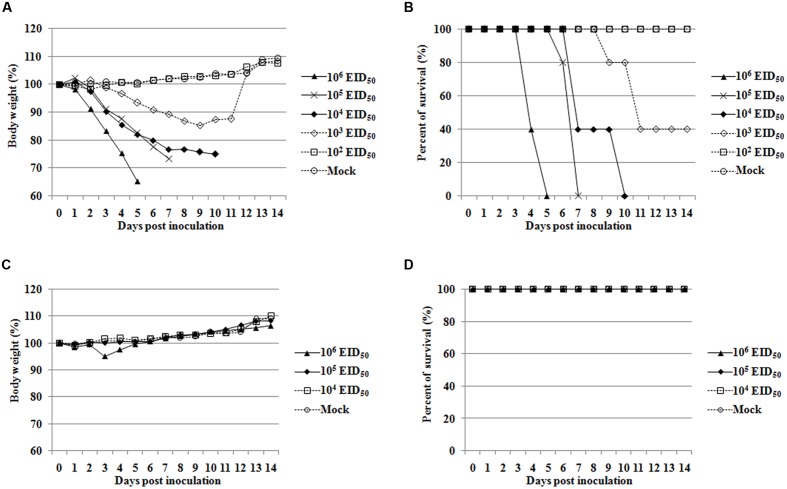
**Pathogenicity of NJ06 and NJ01 viruses in mice.** Groups of five BALB/c mice were inoculated intranasally with different doses of virus (10^2^–10^6^ EID_50_ of NJ06 or 10^4^–10^6^ EID_50_ of NJ01) or mock inoculated (Mock). Morbidity was evaluated by monitoring weight changes over a 14-day period and is graphed as a percentage of the animals’ weight on the day of inoculation (day 0). The average body weight of mice infected with NJ06 **(A)** or NJ01 **(C)** is shown. Mortality of NJ06 **(B)** and NJ01 **(D)** infected mice was examined by measuring the percent survival.

To compare the virulence of NJ06 with that of NJ01 virus, MLD_50_ was determined. The results showed that NJ06 owned an MLD_50_ of 10^2.83^ EID_50_ and was highly pathogenic for mice based on MLD_50_ values of <10^3.0^ EID_50_ (Katz et al., 2000; Chen et al., 2004), while NJ01 was low pathogenicity in mice, with an MLD_50_ of >10^6.81^ EID_50_. The morbidity and mortality of the two viruses were also compared in mice in another study. NJ06-infected mice showed obvious signs of illness, including decreased activity, huddling, ruffled fur, heavy/labored breathing, and hunched posture. Mice in this group began to lose weight at 1 dpi with a high inoculated dose of 10^6.0^ EID_50_, and at 3 dpi with a low inoculated dose of 10^3.0^ EID_50_ (**Figure 1A**). In addition, all of the mice in the NJ06-infected group showed obvious weight loss and died by 5 dpi at a dose of 10^6.0^ EID_50_, and by 10 dpi at a dose of 10^4.0^ EID_50_ (**Figure 1B**). In contrast, no mortality was observed in NJ01-infected mice (**Figure 1D**), and the mice in this group displayed only slight weight reduction throughout the course of infection, and started to gain weight at 5 dpi, even at a high dose of 10^6.0^ EID_50_ (**Figure 1C**).

### Replication of NJ06 and NJ01 Viruses *In vivo* and *In vitro*

**FIGURE 2:**
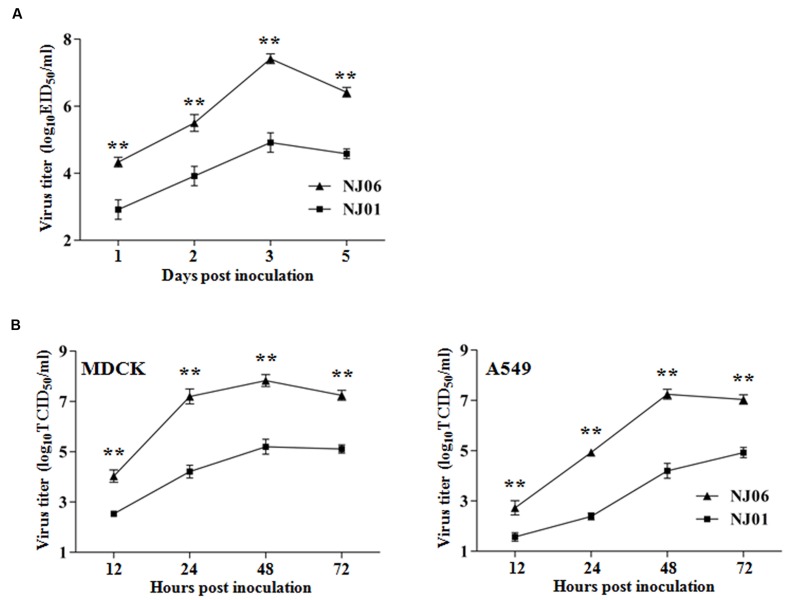
**Replication kinetics of NJ06 and NJ01viruses *in vivo* and *in vitro*.** **(A)** Groups of three BALB/c mice were inoculated intranasally with 10^5^ EID_50_ of NJ06 or NJ01 viruses, and lungs were collected at for virus titration in eggs on the indicated days. **(B)** Madin–Darby canine kidney (MDCK) or A549 cells were infected with NJ06 or NJ01 viruses at a multiplicity of infection of 0.01, and virus yields were determined in MDCK cells at appropriate time points. Data represent the means of the results determined for three independent experiments ± standard deviations. ^∗∗^*P* < 0.01 compared with the value for the NJ01 virus.

To determine whether the differences in virulence of NI06 and NJ01 were related to the differences of viral replication in mice, the levels of viral replication in mouse organs were compared. No infectious virus was detected in the heart, liver, spleen, kidney, brain from any of the NJ06- or NJ01-infected mice, whereas both the two viruses replicated well in mouse lungs. However, the NJ06 virus replicated to a high titer that was 10^1.4^-fold higher than the NJ01 virus as early as 1 dpi and sustained significantly higher levels of replication than the NJ01 virus throughout the course of infection (**Figure 2A**). The NJ06 virus reached a peak titer of 10^7.4^ EID_50_/ml at 3 dpi, versus a peak titer of 10^4.9^ EID_50_/ml at this time point for NJ01. Therefore, the NJ06 virus grew faster and to significantly higher titers than the NJ01 virus, though the two viruses all could replicate in mouse lungs.

The replication kinetics of the two viruses *in vitro* was also measured in MDCK and A549 cells. The NJ06 virus grew to significantly higher titers than NJ01 in either MDCK or A549 cells at each time point (**Figure 2B**). The NJ06 virus reached a peak titer of 10^7.8^ TCID_50_/ml at 48 hpi in MDCK cells, which was 10^2.6^-fold higher than the peak titer of NJ01 at this time point. The peak viral titer of NJ06 virus in A549 cells was also observed on 48 hpi, reaching 10^7.2^ TCID_50_/ml, versus virus titer of 10^4.2^ TCID_50_/ml at 48 hpi and reaching peak yield with 10^4.9^ TCID_50_/ml at 72 hpi for NJ01. Therefore, the replication abilities of NJ06 virus were significantly higher than the NJ01 virus both *in vivo* and *in vitro*, which might correlate with the higher virulence of NJ06 virus in mice.

### Severe Lung Lesions in Mice Infected with NJ06 Virus

**FIGURE 3:**
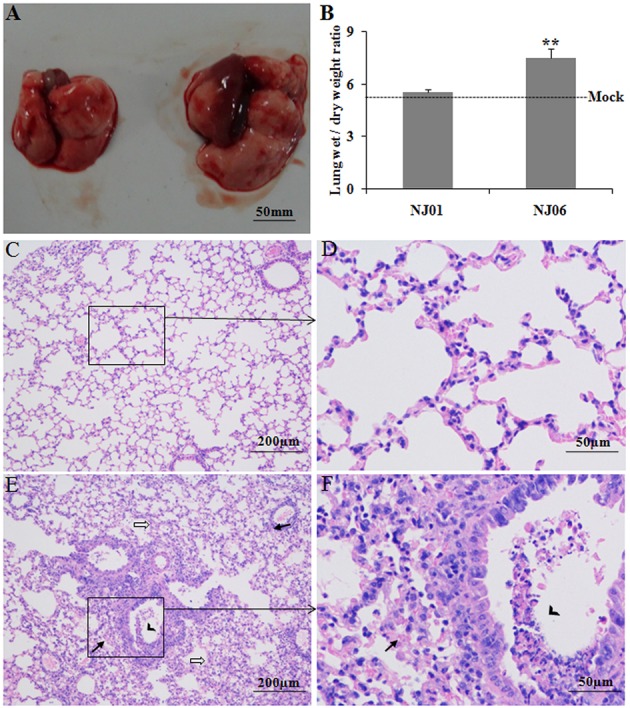
**Lung lesions in mice.** Groups of BALB/c mice were intranasally inoculated with 10^5^ EID_50_ of the NJ06 and NJ01 viruses, respectively, gross pathology, water content, and histopathology of lungs were determined at 5 days post inoculation (dpi). **(A)** Gross pathology of H9N2virus-infected lung. Severe edema, congestion, and hemorrhage were observed in NJ06-infected lung (left), and the NJ01-infected lung (right) appeared normal except for occasional small dark red foci. **(B)** Lung wet/dry weight ratios of H9N2 virus-infected mice. Data represent the means of the results determined for three independent experiments ± standard deviations. ^∗∗^ indicates *p* < 0.01 compared with the mock group. **(C,D)** Representative histopathological changes in NJ01-infected lungs (H&E-stained). Only mild and limited alveolitis was observed. **(E,F)** Representative histopathological changes in NJ06-infected lungs. Solid arrows indicate inflammatory cell infiltrates around the bronchus, open arrows indicate edema and thickening of alveolar walls, Solid arrowheads indicate desquamation of epithelial cells in bronchial lumens.

To compare the lung lesions of mice infected with NJ06 or NJ01, the gross and histopathologic changes in the mouse lung was determined at 5 dpi, a time that immediately preceded the death of mice infected with the NJ06 virus. We found that NJ06-infected mice exhibited severe edema, congestion, and hemorrhage in lungs (**Figure 3A**, right), whereas the lung of NJ01-infected mice appeared normal except for occasional small dark red foci of pneumonia (**Figure 3A**, left). Moreover, the severe pulmonary edema in NJ06-infected mice was further confirmed by the lung wet/dry weight ratios (**Figure 3B**). Histologically, NJ06 induced severe pneumonia with inflammatory cellular infiltration and hemorrhage, alveolar wall edema and thickening, and deciduous epithelium mucosae and inflammatory cells in the bronchioles (**Figures 3C,D**). However, only mild and limited alveolitis was observed in the lungs of NJ01-infected mice (**Figures 3E,F**).

### Increased Numbers of BAL Cells in Mice Infected with NJ06 Virus

**FIGURE 4:**
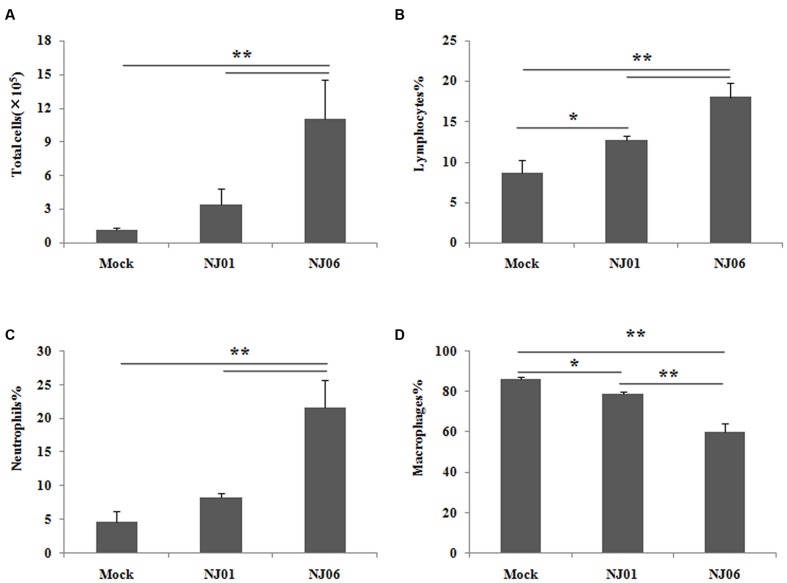
**Total and differential cell counts in bronchoalveolar lavage (BAL) fluid.** Groups of five mice were inoculated intranasally with 10^5^ EID_50_ of NJ06 or NJ01 virus, and BAL specimens were collected to determine cell counts on 5 dpi. The total numbers of BAL cells **(A)** and percentages of lymphocytes **(B)**, neutrophils, **(C)** and macrophages **(D)** of virus- or mock-inoculated mice are shown as means ± SD. ^∗^*p* < 0.05 and ^∗∗^*p* < 0.01.

To better characterize the inflammatory cellular components in lungs, total and differential cell counts in BAL fluid were determined at 5 dpi for NJ06- and NJ01-infected mice, respectively. The total number of BAL cells was markedly increased in mice infected with NJ06 virus compared to the NJ01 virus or mock infection group (**Figure 4A**). By contrast, there were no statistically significant differences in the total numbers of BAL cells between the NJ01 and mock infection groups. In addition, cellular infiltration in the BAL samples during NJ06 virus infection was associated with an increase in the percentage of neutrophils and lymphocytes compared with that for NJ01-infected mice (**Figures 4B–D**). These data suggested that NJ06 virus induced a much larger increase of inflammatory cell infiltrate into the lungs, especially neutrophils and lymphocytes, which may be contributing to the pathogenesis of the severe lung injury associated with NJ06 virus infection.

### NJ06 Virus Elicits Significantly High Levels of Cytokine Response in Mouse Lungs

**FIGURE 5:**
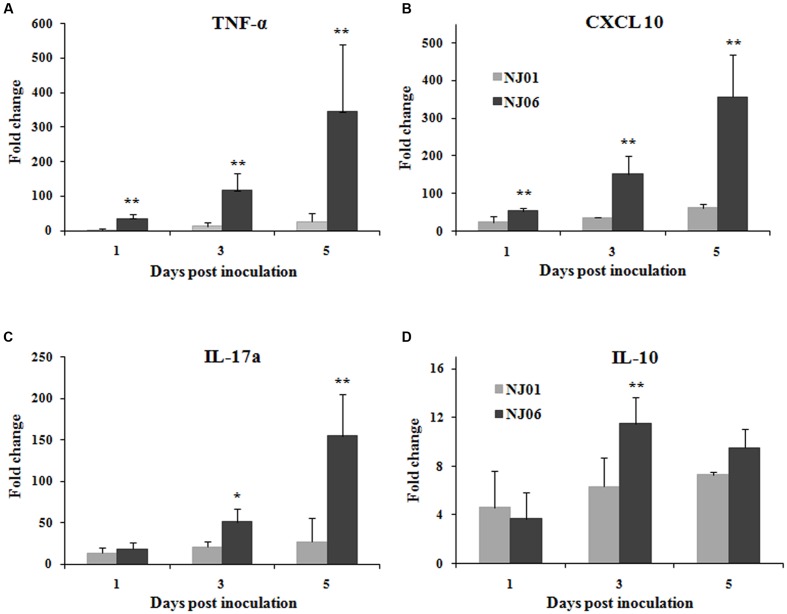
**Inflammatory cytokines response in mouse lungs.** Groups of five mice were intranasally inoculated with 10^5^ ELD_50_ of NJ06 or NJ01 virus, and the expression of these cytokines, TNF-α, IL-1β, IL-8, and IL-10, was measured in the mouse lungs at the indicated times by Quantitative real-time PCR (qRT-PCR). Each cytokine expression was normalized to the expression of β-actin, and is presented as the fold change relative to the level of the mock group. The mean fold change of each group is shown, with error bars representing the SD. ^∗^ indicates *p* < 0.05 and ^∗∗^ indicates *p* < 0.01 compared with the NJ01 virus infection group.

To determine whether the different levels of virulence of NJ06 and NJ01 viruses were related to difference in cytokine expression levels induced by the two viruses in mice, lungs from mice infected with 10^5^ EID_50_ of NJ06 or NJ01 virus were collected, and subsequently assayed for TNF-α, CXCL10, IL-17a, and IL-10 levels by qRT-PCR, respectively. The levels of all cytokines were substantially greater than constitutive levels in the lungs of NJ06- or NJ01-infected mice by 1 dpi (**Figure 5**). However, NJ06 virus induced significantly higher levels of TNF-α and CXCL 10 expression than did NJ01 at all time points. The IL-17a was also detected at higher levels of expression in NJ06-infected mice than in NJ01-infected mice, although the result at the early time point, 1 dpi, was not significant. By contrast, at 1 dpi, the levels of IL-10 were reduced in NJ06-infected mice compared with those in NJ01-infected mice. Although the levels of IL-10 were elevated in NJ06-infected mice at 3 and 5 dpi, the latter result was not significant compared with levels found in NJ01-infected mice. In addition, a continuous increase in levels of IL-10 was observed throughout the entire study period in NJ01-infected mice, whereas the IL-10 levels in NJ06-infected mice were reduced at the end of the observational period (5 dpi) compared with the former time point (3 dpi). These data suggest that the induction of inflammatory cytokines by NJ06 is different from the induction by NJ06, which might contribute to the observed differences in severity of disease caused by the two viruses.

### Sequence and Phylogenetic Analysis

**FIGURE 6:**
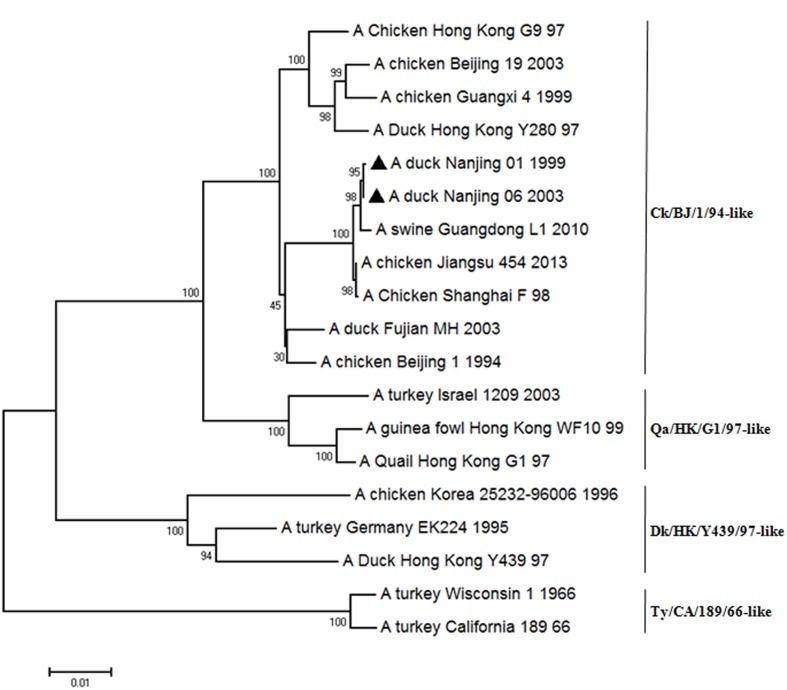
**Phylogenetic tree of HA genes of representative H9N2 influenza A viruses.** The tree was generated by the distance-based neighbor-joining method using software MEGA 6.06. The reliability of the tree was assessed by bootstrap analysis with 1000 replicates. The viruses tested in this study are marked with black triangle.

To determine the molecular basis for the differences in pathogenicity between the two viruses, the sequences of all of eight segments of NJ06 were compared with those of NJ01 virus. This revealed twelve amino acid differences between these two viruses, which were mapped to PB2, PB1, PA, HA (H3 numbering used throughout the text), NP, NA, and NS gene (**Table 1**). Phylogenetic analysis of the HA genes showed that both viruses belonged to the Ck/BJ/1/94-like lineage (**Figure 6**), with the same R-S-S-R amino acid motif at the cleavage sites, a characteristic of low pathogenic avian influenza virus (LPAIV) between HA1 and HA2 [4,34]. The NA and M genes of these two isolates also belong to the Ck/BJ/1/94-like lineage (**Supplementary Figure S1**), and both viruses had the same “marking” deletion of three amino acids (positions 62–64) at the NA stalk region, as previously described (9, 11, 15). The NS and ribonucleoprotein (PB2, PB1, PA, and NP) complex genes of the two viruses fell into the DK/HK/Y439/97-like and Ck/SH/F/98-like, respectively (**Supplementary Figure S1**).

**Table 1:** Amino acid differences between the NJ06 and NJ01 viruses.

Viruses	Amino acid residue at indicated position (Number of strains possessing mutation/total number of strains examined)								
	
	PB2		PB1	PA		HA	NP			NA		NS1
							
	149	627	187	548	550	157^∗^	127	277	340	9	435	171
NJ 06	T	K	R	L	M	K	G	P	D	A	R	D
NJ 01	P	E	K	M	L	E	E	H	N	T	K	N
Avian	P(1099/1100)	E(1018/1100)	R(1106/1108)	M(1164/1168)	L(1119/1168)	K(2017/2032)	E(1178/1182)	P(1181/1182)	D(1178/1182)	A(1210/1431)	R(1392/1431)	D(1339/1663)
	S(1/1100)	V(74/1100)	K(2/1108)	I(3/1168)	I(49/1168)	N(3/2032)	D(2/1182)	H(1/1182)	G(1/1182)	T(192/1431)	K(26/1431)	N(93/1663)
		K(6/1100)		X(1/1168)		Q(3/2032)	K(1/1182)		E(1/1182)	V(15/1431)	I(7/1431)	T(64/1663)
		A(1/1100)				R(2/2032)	G(1/1182)		H(1/1182)	S(7/1431)	S(3/1431)	E(54/1663)
		G(1/1100)				X(2/2032)			N(1/1182)	P(2/1431)	G(3/1431)	Y(39/1663)
						E(2/2032)				I(2/1431)		G(11/1663)
						T(2/2032)				E(1/1431)		A(1/1663)
						D(1/2032)				F(1/1431)		H(1/1663)
										Y(1/1431)		V(1/1663)
Swine	P(28/28)	E(39/39)	R(28/30)	M(31/31)	L(31/31)	K(43/44)	E(31/32)	P(35/35)	D(35/35)	A(29/32)	R(41/43)	D(40/42)
			K(2/30)			N(1/44)	D(1/32)			T(3/32)	K(2/43)	N(2/42)
Human	P(14/14)	E(13/13)	R(14/14)	M(14/14)	L(14/14)	K(21/21)	E(14/14)	P(13/13)	D(13/13)	A(18/18)	R(20/20)	D(11/14)
												E(3/14)


*^∗^H3 numbering.*

To find out whether the amino acids found at these positions in NJ06 were present also in other natural H9N2 strains, we analyzed the H9N2 sequences deposited in the Influenza Research Database (the National Institute of Allergy and Infectious Diseases database^1^; **Table 1**). Most avian, swine, and human isolates possess the same amino acid as NJ06 at positions PB1-187 (R), HA-147 and -477 (K and Y), NP-277 and -340 (P and D), NA-9 and -435 (A and R), and NS1-171(D). By contrast, at positions PB2-149 and -627, PA-548 and -550, and NP-127, most avian, swine, and human isolates share common residues as NJ01 virus. In fact, only five and one avian isolates share the same residues with the NJ06 virus at positions PB2-627(K) and NP-127 (G), respectively, and no isolates was found to contain the residues PB2-149T, and PA-548L and -550M as NJ06 virus. Therefore, the amino acids observed at these positions in NJ06 were unique to this virus, which might contribute to the high virulence of the NJ06 virus.

## Discussion

Although highly pathogenic avian influenza viruses (HPAIVs), such as H5 and H7 viruses, have caused serious harm to human health, some recent studies have suggested that LPAIVs, especially H9N2 viruses, could jump to humans more easily (Wan and Perez, 2007; Long et al., 2015). H9N2 AIVs have repeatedly infected humans and other mammals (Peiris et al., 1999; Yu et al., 2011; Sun et al., 2013), such as pigs and dogs, and could cause mild respiratory disease in humans (Guo et al., 1999; Peiris et al., 1999). More seriously, some H9N2 AIVs isolates could replicate efficiently in mice and ferrets without prior adaptation, and was able to adapt to high pathogenicity in mice. All these facts indicate that H9N2 AIVs have gradually acquired mutations that make them more adapted to mammals including humans (Wan and Perez, 2007; Kimble et al., 2011; Mok et al., 2011), posing a significant threat to public health. Therefore, it is necessary to investigate the pathogenesis of H9N2 AIVs in mammals.

Although the ferret is well established as an animal model to study human influenza virus pathogenesis and transmission, no animal model is perfect and the use of ferrets for influenza studies has been limited by the lack of availability of inbred and specific pathogen–free animals, and the corresponding immunological reagents (Oh and Hurt, 2016). Therefore, the mouse, another commonly used model in influenza virus research (Belser and Tumpey, 2013; Cauldwell et al., 2014; Thangavel and Bouvier, 2014), was used to compared the pathogenicity of the two genetically similar H9N2 viruses in this study. We found that the NJ06 virus was highly pathogenic for mice, while the NJ01 virus exhibited low pathogenicity in this animal model. The NJ06 virus caused signs of severe disease and resulted in 60% mortality at a low inoculation dose of 10^3^ EID_50_, whereas infection with the DK1 virus did not cause death or obvious clinical signs of illness even at a high dose of 10^6^ EID_50_. Previous studies showed that the high virulence of H5N1 AIVs for mice is associated with the enhanced replication and extra-pulmonary infection (Sirinonthanawech et al., 2011; Hu et al., 2013). Here, the NJ06 virus grew faster and to significantly higher titers in mouse lungs than NJ01 virus, but both the two viruses were not able to spread to the extra-pulmonary organs. These data support the viewpoint that high replication ability replication in lungs is an important and characteristic prerequisite for high virulence of AIV in mice.

Severe lung lesions characterized by massive edema, diffuse alveolar damage, and excessive inflammatory cell infiltration are involved in the severe influenza in humans and animal models caused by avian viruses, such as H5N1 and H7N9, or highly pathogenic human viruses, such as the 1918 H1N1 virus (Gambotto et al., 2008; Franco-Paredes et al., 2009; Uyeki, 2009; Zheng et al., 2013; Feng et al., 2014; Li et al., 2014; Hrincius et al., 2015). Our results showed that the NJ06 infection could result in severe edema and alveolar damage, and elevated inflammatory cell infiltration in mouse lungs, whereas no difference in lung water content was observed between the NJ01-infected group and the control group, and only mild and limited alveolitis was observed in the NJ01-infected lungs. In addition, the NJ06 infection resulted in significantly higher numbers of inflammatory cells in BAL than NJ01 or mock infection. Furthermore, the percentages of neutrophils in BAL cells in NJ06-infected mice were significantly higher than those in NJ01-infected mice. Neutrophils are primary mediator/effector cells involved in producing acute lung injury (Headley et al., 1997; Ayala et al., 2002), and the elevated levels of neutrophils have also been found in the BAL samples of mice infected with highly pathogenic H5N1 viruses or the novel H7N9 viruses (Xu et al., 2009, 2013; Feng et al., 2015). Therefore, enhanced pulmonary neutrophil invasion may be associated with the severity of NJ06 virus infection in mice.

It is generally accepted that dysregulation of cytokine response is associated with the high virulence of AIVs in mammals (Us, 2008). As expected, the NJ06 virus caused intense expression of proinflammatory cytokine genes, such as TNF-α, CXCL10, and IL-17a. TNF-α is a key factor modulating neutrophil activity, and a high level of this cytokine has been linked to the hyperresponsiveness of neutrophils (Grommes and Soehnlein, 2011). CXCL10 is a potent chemoattractant for activated Th1 lymphocytes and natural killer cells and plays a role in the temporal development of innate and adaptive immunity in concert with type I and II IFNs (Neville et al., 1997). The high levels of TNF-α and CXCL10 have been linked to the persistent severe viral disease in patients with severe acute respiratory syndrome (Tobinick, 2004; de Jong et al., 2006). IL-17a acts as a pro-inflammatory cytokine that induces the expansion and accumulation of neutrophils of the innate immune system (Perrone et al., 2008; Crowe et al., 2009) and plays a critical role in mediating the acute lung injury caused by 2009 pandemic H1N1 influenza infection (Li et al., 2012). Therefore, based on the established role of these cytokines in viral disease, our results suggest that these pro-inflammatory cytokines may have pathological importance in NJ06 infection and are partially responsible for disease pathogenesis.

Although the NJ06 virus showed higher virulence and induced more severe lung injury in mice compared with the NJ01virus, the two viruses differed only by 12 amino acids distributed throughout seven genes. Except for PB2-E627K, none of these amino acid differences had been recognized to be related to increased virulence or replication efficiency. The amino acid at position 627 of PB2 is a well known determinant of host range, and the substitution E627K has been shown to be crucial for the adaptation and increased virulence of avian influenza viruses in mammals (Subbarao et al., 1993; Hatta et al., 2001; Fornek et al., 2009; Li et al., 2009). However, H5N1 isolates with PB2 627E are also lethal to human (Mehle and Doudna, 2009) and mouse, whereas H9N2 isolates with PB2 627K are not lethal for mouse (Wang et al., 2012; Liu et al., 2014), indicating that the PB2 627K is not a sole determinant factor for mammalian adaptation by avian influenza viruses or its contribution to virulence need to interact with residue at other positions or genes (Wang et al., 2012; Liu et al., 2015). In addition, all other ten differences, except for NA-K435R, were located in the recognized functional regions that are involved in interaction of viral proteins or between the virus and host factors. The HA-E157K residues locate to the antigenic site I of the H9 HA corresponding to site B in H3 HA (Both et al., 1983; Kaverin et al., 2004), and the NA-T9A residues located in the amino-terminal transmembrane domain (Shtyrya et al., 2009). The NS-N171D residues resides in the host cleavage and polyadenylation factor (CPSF30) binding domain (Nemeroff et al., 1998), which has been found to be associated with the inhibition of 3′-end processing of cellular pre-mRNAs, including IFN-β pre-mRNA (Noah et al., 2003). Seven different residues (PB2-P149T, PB2-E627K, PB1-K187R, PA-M548L, PA-L550M, NP-E127G, NP-H277P, and NP-N340D) in the RNP complexes were all located in the known functional regions, including the NP binding domain of the PB2 protein, the nuclear localization sequence (NLS) of the PB1 protein, the PB1 binding domain of the PA protein, and the PB2 binding regions of the NP protein (Liu et al., 2009; Ng et al., 2009; Boivin et al., 2010; Ping et al., 2011). Therefore, besides PB2 E627K, other residue differences may also be associated with the high virulence of the NJ06 virus.

In summary, our study showed that a natural H9N2 isolate is highly pathogenic for mice, which might suggest the potential threat of H9N2 AIVs for other mammals, including humans, and also highlight the necessity for continued evaluation of the viral pathogenicity for mammals in the surveillance for LPAIVs, especially H9N2 viruses. In addition, comparison of the predicted amino acid sequences of NJ06 and NJ01 viruses showed that twelve residues differences in specific functional regions of viral genome resulted in the highly pathogenic phenotypes of the NJ06 virus, including rapid growth *in vivo* and *in vitro*, severe pulmonary lesions, excessive inflammatory cellular infiltration and cytokine response in lungs, and death in mice. However, it is not yet known which residues differences, or combinations of differences, are responsible for the high virulence for mice. We are currently attempting to determine the role of individual mutations in the viruses’ pathogenicity in mice using a reverse genetics approach.

## Author Contributions

YiL and QL conceived and designed this research; QL, YuL, JY, and XH performed this experiments; DZ and KH analyzed the data; KB contributed reagents/analytic tools; QL and YuL wrote the paper. All authors read and approved the paper.

## Conflict of Interest Statement

The authors declare that the research was conducted in the absence of any commercial or financial relationships that could be construed as a potential conflict of interest.

## Footnotes

1 http://www.fludb.org
