# Supplementary material for: Two Genetically Similar H9N2 Influenza A Viruses Show Different Pathogenicity in Mice
Source: Front Microbiol. 2016 Nov 4;7:1737. doi: 10.3389/fmicb.2016.01737 (PMC5096341; doi:10.3389/fmicb.2016.01737)
Supplement: Supplementary file 1 [file Presentation_1.ZIP › Figure S1/Figur legends for S1.docx]

**Figure S1 Phylogenetic tree of NA, M, NS, NP, PB2, PB1 and PA genes of representative H9N2 influenza A viruses.** The tree was generated by the distance-based neighbor-joining method using software MEGA 6.06. The reliability of the tree was assessed by bootstrap analysis with 1000 replicates. The viruses tested in this study are marked with black triangle.
