# Supplementary figures and images for: Two Genetically Similar H9N2 Influenza A Viruses Show Different Pathogenicity in Mice
Source: Front Microbiol. 2016 Nov 4;7:1737. doi: 10.3389/fmicb.2016.01737 (PMC5096341; doi:10.3389/fmicb.2016.01737)

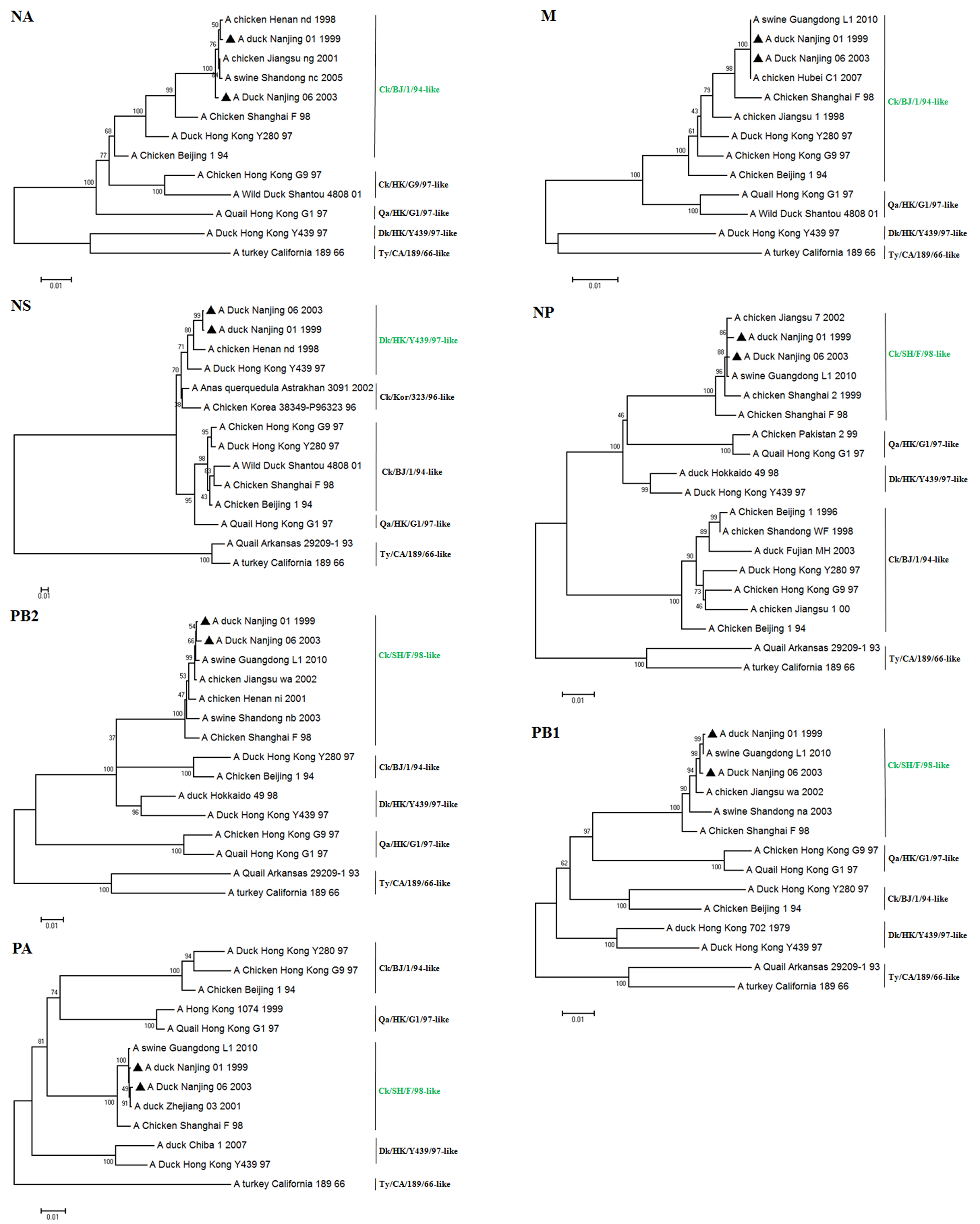

Supplement: Supplementary file 1 [file Presentation_1.ZIP › Figure S1/Figure S1.tif]
